# FODMAP Diet in Celiac Disease and Gluten-Related Disorders

**DOI:** 10.3390/nu16234190

**Published:** 2024-12-04

**Authors:** Paolo Usai Satta, Giammarco Mocci, Mariantonia Lai

**Affiliations:** 1 Gastroenterology Unit, ARNAS G. Brotzu, 09121 Cagliari, Italy; giammarco.mocci@gmail.com; 2 Digestive Endoscopy, ARNAS G. Brotzu, 09121 Cagliari, Italy; laimariantonia@gmail.com

**Keywords:** celiac disease, non-celiac gluten/wheat sensitivity, gluten-free diet, FODMAP diet, low-FODMAP diet

## Abstract

Background: Individuals with celiac disease (CD) often report the persistence of gastrointestinal symptoms despite adherence to a gluten-free diet (GFD). A diet rich in fermentable oligo-, di-, and monosaccharides and polyols (FODMAPs) could cause symptoms in CD on a GFD, and conversely a low-FODMAP diet could positively influence the therapeutic management of CD and non-celiac gluten sensitivity (NCGS). The aim of this review was to explore the hypothetical impact of the FODMAD diet and the low-FODMAP diet (LFD) in CD and gluten-related disorders. Methods: A complete online search for FODMAP related to CD, NCGS, and the GFD was carried out using the Pubmed, Medline, and Cochrane databases. Results: Indeed, an LFD could successfully provide symptom relief in GFD-treated CD patients. Fructans, typical components of FODMAPs, have been associated with digestive symptoms in NCGS, and an LFD could improve the clinical picture. According to some evidence, an LFD could also improve the psychological status both in celiac patients on a GFD and in NCGS. However, an LFD should not have a significant impact on gut microbiota. Conclusions: Recent evidence supports the role of FODMAP restriction in CD patients with persistent symptoms on a GFD and in decreasing gastrointestinal disturbances in NCGS, although the GFD still represents the first-line therapy.

## 1. Introduction

Celiac disease (CD) is a chronic, gluten-dependent disease characterized by small bowel mucosal inflammation and injury and consequent malabsorption in genetically predisposed subjects [1,2,3]. In the absence of clinical and diagnostic evidence of CD, self-reported gluten/wheat sensitivity has recently been defined as non-celiac gluten/wheat sensitivity (NCGWS) [4]. On the other hand, there is no specific diagnostic test to detect NCGWS. A gluten-free diet (GFD) is the only available therapeutic approach to improve or normalize these disorders [1,2,3]. It is notable that chronic digestive symptoms compatible with irritable bowel disease (IBS) may be experienced by up to 50% of celiac patients, despite adherence to a GFD [5]. Recently, dietary and lifestyle modifications have been considered as an effective therapeutic approach for IBS patients. In detail, a limited period with a diet low in fermentable oligo-, di-, and monosaccharides and polyols (FODMAPs) has been proposed in patients with IBS [6]. A diet low in FODMAP components could also influence the therapeutic management of CD and NCGS patients. The aim of this review was to explore the hypothetical impact of FODMAD intake and the low-FODMAP diet (LFD) in CD and gluten-related disorders.

## 2. Materials and Methods

A complete online search of Pubmed and EMBASE was carried out using the keywords “celiac disease”, “non celiac gluten/wheat sensitivity”, “FODMAP diet”, and “gluten related disorders” in various combinations with the Boolean operators “and”, “or”, and “not”. The search generally included articles related to human studies. We performed manual cross-referencing and selected articles published in English between January 2014 and October 2024.

## 3. FODMAPs

### 3.1. FODMAP Components

FODMAPs include several dietary components. Among carbohydrates, lactose and dairy products are among the most important components. Fructose is found in honey, apples, and pears; fructans in wheat, garlic, onion, and rye; and galactans in legumes and cabbage. In addition, there are polyols, which include sorbitol and mannitol. Polyols can be found in certain fruits, vegetables, mushrooms, and artificial sweeteners [7]. In addition, fructans and galacto-oligosaccharides have a typical prebiotic action. Prebiotics are non-digestible dietary fibers that favor the presence of positive bacterial genera in the digestive tract. Non-digestible alimentary carbohydrates are also commonly employed in the alimentary distribution [8]. The typical behavior of these ingredients is to be scarcely absorbed in the small bowel. Malabsorption of lactose and fructose is directly caused by a reduced or absent digestive enzymatic action or compromised transport of carbohydrates across the intestinal barrier. In cases of malabsorption, FODMAPs exert a significant osmotic effect, and they are rapidly fermented by bacteria in the digestive tract. Consequently, patients complain of abdominal pain, diarrhea, and bloating, which are typical symptoms present in IBS.

### 3.2. FODMAPs, IBS, and Bowel Diseases

Data from several studies support the role of an LFD in the management of IBS patients [9,10,11]. In fact, several studies have consistently shown symptomatic benefits of an LFD in most patients with IBS [6]. However, one study that compared the diet commonly used in these patients with an LFD did not demonstrate significant differences in symptom improvement [12]. In addition, the utility of an LFD has been evaluated in other digestive diseases with conflicting results. Pedersen et al. showed clinical improvement of persistent digestive symptoms in inflammatory bowel disease (IBD) [13], while Cox et al. showed worsened digestive symptoms in patients with IBD [14]. On the other hand, in a study by Testa et al. an LFD was shown to be a useful option to control abdominal symptoms, improving quality of life (QoL), in patients with IBS, non-active IBD, or CD on a GFD [15].

## 4. FODMAP Diet and Celiac Disease

### 4.1. Persistent Digestive Symptoms in Celiac Disease

According to clinical experience, many celiac patients experience persistent digestive symptoms despite long-term adherence to a GFD [5,16,17]. There are several causes that lead to presumed nonresponsive CD, including the persistence of gluten exposure, small intestinal bacterial overgrowth, microscopic colitis, IBS, and refractory CD. Furthermore, the persistence of digestive symptoms may cause poor dietary adherence in celiac patients on a GFD. Awareness of these clinical findings is fundamental to improving the clinical management and follow-up of celiac patients, and refractory CD must first be excluded. In detail, a higher frequence of IBS symptoms in celiac patients on a GFD than in the general population has been suggested by several studies [18,19], but the actual prevalence of functional gut disorders in CD patients remains controversial. A study by Barratt et al. [20] showed that IBS is more frequent in CD patients on a GFD than in age-matched and sex-matched controls. A meta-analysis by Bansal et al. [21] showed a pooled prevalence of IBS symptoms of 38% (95% CI: 27.0–50.0%) in all patients with CD. Moreover, functional gastrointestinal disorders are associated with health impairments and psychological problems in CD [22,23,24].

### 4.2. FODMAPs and Celiac Disease

The relationship between persistent digestive symptoms in CD on a GFD and FODMAP intake was evaluated in two studies (one in pediatric patients and the other in adult subjects) [25,26], as reported in a recent systematic review [27]. In celiac patients on a GFD, FODMAP intake was lower than in healthy controls in both studies. However, digestive symptoms and FODMAP intake were not directly related. Among the FODMAP components, higher consumption of fruits and vegetables was recorded in the CD subjects, whereas cereals and sweets with high FODMAP contents were consumed by the non-celiac controls. On the other hand, two RCTs and two open-label interventional studies explored the clinical impact of an LFD and a GFD compared to a simple GFD in CD patients with persistent digestive symptoms. All studies included CD patients in serologic [15,28] or histologic remission [29,30], with persistent IBS-like symptoms despite adequate adherence to a GFD. In detail, 176 adult CD patients (86 females) were recruited, and 115 followed an LFD and GFD protocol. All studies agreed on the ability of an LFD-GFD to improve digestive symptoms in these patients. To assess the clinical efficacy of this dietary approach, the Gastrointestinal Symptom Rating Scale (GSRS) was used in two studies [27,30], whereas the other two studies used the IBS severity scoring system (IBS-SSS) [15] and a visual analogic scale (VAS) [26]. Symptoms measured by the GSRS significantly improved following adherence to an LFD and a GFD for 4 weeks compared to a simple GFD in the first study (*p* < 0.001) [28], and symptoms improved compared to the baseline digestive symptoms in the second study (*p* = 0.007) [28]. Similarly, Roncoroni et al. found that abdominal pain was reduced after 21 days of an LFD and a GFD compared to a simple GFD (*p* < 0.01) [29]. In addition, digestive symptoms, established with the IBS-SSS scoring system by Testa et al., improved after 1 and 3 months of an LFD in all patients with CD. Moreover, two studies evaluated the impact of an LFD on QoL in celiac patients. More precisely, Roncoroni et al. highlighted significant improvements in psychological symptoms and general well-being after 21 days of an LFD in comparison with the control group. Similarly, in the study by Testa et al., QoL was improved after the dietary intervention of a period of 3 months on an LFD in IBS patients, IBD patients in remission, and CD patients, although there was no statistical difference [15]. In any case, dieticians have a fundamental role in managing personalized and balanced LFDs to avoid nutritional consequences in CD patients. Finally, specific attention should be given to the risk that an LFD could affect the gut microbiota in CD patients with persistent symptoms on a GFD. In a recent study [31], although the fecal microbiota changed more in the LFD group than in the control group, these findings did not result in any difference in bacterial α-diversity between the groups at follow-up. The relatively modest effects of the LFD on the gut microbiota in CD patients are encouraging with respect to LFD safety.

## 5. FODMAP Diet and Non-Celiac Gluten/Wheat Sensitivity

### 5.1. Non-Celiac Gluten/Wheat Sensitivity

Typical symptoms related to NCGS are diarrhea, bloating, and abdominal pain. IBS with diarrhea can overlap and can be clinically similar to NCGS [5]. The main difference between NCGS and IBS is that patients with NCGS self-report symptoms when consuming gluten. On the contrary, IBS patients generally do not report gluten ingestion as a specific trigger for their symptoms [32]. However, up to 80% of IBS patients complain of postprandial symptoms, and generic food represents a typical provocative stimulus. Furthermore, many patients presume they have food intolerances [32,33]. However, in addition to gluten proteins, other wheat-related components may provoke the symptoms that occur in patients with NCGS. Therefore, the term non-celiac wheat sensitivity (NCWS) has in fact replaced the original non-celiac gluten sensitivity [34,35].

### 5.2. FODMAPs and NCGWS

Wheat contains several non-gluten components that could provoke digestive discomfort. In detail, fructans are typical non-gluten dietary components related to FODMAPs [34,35]. However, the mechanism by which gluten and wheat cause IBS-like symptoms is not clear [36]. In a study using confocal endomicroscopy, the integrity of the small intestinal mucosa was affected by wheat administered endoscopically into the duodenal mucosa [37]. In another study, a gluten challenge increased gut permeability in a group of NCGS patients and IBS patients with diarrhea [38]. A recent systematic review selected three randomized clinical trials that aimed to explore the potential relationship between FODMAPs and NCGS [39]. These studies used different methods to evaluate clinical outcomes related to gastrointestinal symptoms. In detail, Biesiekiesrki et al. [40] utilized a VAS, Dieterich et al. [41] the GSRS for IBS, and Skodje et al. both methods [42]. Abdominal pain and bloating were associated with a fructan challenge in the double-blind, placebo-controlled study by Skodje et al. involving 59 subjects with self-reported NCGS. Using a VAS, Biesiekierski et al. also found that FODMAPs and, more specifically, fructans may cause gastrointestinal symptoms in NCGS patients. Using the GSRS for IBS, Dieterich et al. and Skodje et al. showed that an LFD was associated with less severe gastrointestinal symptoms, particularly abdominal pain. The same three studies measured psychological symptoms and QoL with different instruments and scales that were hardly comparable with each other. In general, the studies were unanimous in showing that dietary intake of FODMAPs worsened psychological symptoms at baseline. The LFD was associated with improved psychological symptoms in the studies by Dieterich et al. and Biesiekierski et al. compared with the GFD. Finally, a recent randomized clinical trial explored the impact of fructans on gut microbiota in NCGS patients [43]. In detail, increased abdominal pain was related to a reduction in the E. coprostanoligenes group after the fructan challenge. In addition, following a gluten challenge and the introduction of fructans, digestive symptoms were linked to variable bacterial abundances. Compared to gluten and placebo challenges, significant changes following the introduction of fructans affected four taxa belonging to the Clostridiales order. Ruminococcaceae, Fusicatenibacter, the Eubacterium coprostanoligenes group, and Anaerotruncus were the considered genera. After the fructan challenge, an abundance of Fusicatenibacter was recorded, whereas the genera Ruminococcaceae, E. coprostanoligenes, and Anaerotruncus were reduced. Finally, no differences in the mean changes were found between the gluten and placebo challenges for these four taxa.

## 6. Conclusions

Clinical practice suggests that some CD patients have persistent digestive symptoms despite adherence to a GFD. The presence of IBS could explain the persistence of digestive disturbances in celiac patients on a regular GFD. An LFD is commonly used for the treatment of IBS. Some evidence indicates that the efficacy of the LFD extends beyond IBS. Higher consumption of vegetables and fruits with high FODMAP contents has been reported in celiac patients, although a clinical impact of a high-FODMAP diet is not proven. However, an LFD can decrease gastrointestinal disturbances in CD patients on a GFD and improve their QoL. Several studies showed that FODMAPs, especially fructans, can cause digestive symptoms in NCGCS. An LFD was associated with less severe gastrointestinal symptoms, particularly abdominal pain, and may improve psychological symptoms in NCGS patients. Table 1 summarizes the more significant studies evaluating the efficacy of an LFD in celiac patients and non-celiac gluten/wheat-sensitive subjects. In conclusion, patients with CD and NCGCS often have comorbidities and persistent symptoms that require personalized management, including nutritional interventions.

## Figures and Tables

**Table 1:** A summary of the most significant studies on the effects of an LFD in celiac disease and non-celiac gluten/wheat sensitivity.

Authors (Ref.)	Study Design	Study Duration	Participants	Diet Methods	Results
Roncoroni [29]	RCT	21 days	50 CD	LFD-GFD vs. GFD alone	LFD improved digestive and psychological symptoms.
Van Megen [28]	Open-label study	4 weeks	70 CD	LFD-GFD vs. GFD alone	Symptoms improved by week 4.
Testa [15]	RCT	3 months	127 pts: 56 IBS, 30 IBD, 41 CD	LFD-GFD for CD pts	Symptoms improved after 1 and 3 months.
Trott [30]	Open-label prospective study	4 weeks	15 CD	LFD	Symptoms improved after 4 weeks.
Dieterich [41]	RCT	8 weeks and 5 days	29 NCGS	10 g of gluten and LFD	LFD improved reflux, abdominal pain, and psychological symptoms.
Biesiekierski [40]	DBP trial	2 weeks of LFD, followed by 1 week of low gluten, high gluten, or placebo	37 IBS-NCGS	High- and low-gluten challenges and LFD	Gluten had no effect on IBS symptoms. Only LFD had effect.
Skodje [42]	DBP trial	6 weeks	59 NCWS	Fructan challenge and LFD	Fructans worsened digestive symptoms. LFD decreased abdominal pain.

Notes: DBP: double-blind placebo-controlled; RCT: randomized clinical trial; CD: celiac disease; IBS: irritable bowel syndrome; IBD: inflammatory bowel disease; GFD: gluten-free diet; LFD: low-FODMAP diet; NCGS: non-celiac gluten sensitivity.

## Data Availability

The original contributions presented in the study are included in the article, further inquiries can be directed to the corresponding author.
